# A discrepancy of penile hemodynamics during visual sexual stimulation observed by near-infrared spectroscopy

**DOI:** 10.1186/s12894-015-0005-x

**Published:** 2015-02-21

**Authors:** Evgenii Kim, Songhyun Lee, Zephaniah Phillips, Jae G Kim

**Affiliations:** School of Information and Communications, Gwangju Institute of Science and Technology, Gwangju, 500-712 Korea; Department of Medical System Engineering, Gwangju Institute of Science and Technology, Gwangju, 500-712 Korea

**Keywords:** NIRS, Hemodynamic, Erectile dysfunction, Penile erection

## Abstract

**Background:**

In this paper, we observed a discrepancy of penile hemodynamics dependent on location by using near infrared spectroscopy (NIRS) sensor, and showcase NIRS as a potentially suitable sensor in supplementing the diagnosis and treatment of erectile dysfunction.

**Methods:**

To observe the effect that location has on penile hemodynamics, the NIRS sensor was placed on the top and the side of genital organ, and oxy- (HbO), deoxy-(RHb), and total (HbT) hemoglobin concentration changes were acquired. Our results from 6 healthy subjects show that hemodynamic changes vary depending on where the probe was placed. To observe a statistical difference between the signals, a Wilcoxon signed-rank test was performed.

**Results:**

The result shows a significant difference (*p* < 0.05) between concentration changes of RHb and HbT depending on the probes’ location. Moreover, the sensor placed on the top of the organ shows a rise of HbO and HbT concentration while RHb concentration decreased. However, hemodynamics from the side of the organ showed that RHb concentration increased along with HbO.

**Conclusions:**

The outcomes demonstrates an ability of NIRS to be sensitive enough to detect the different hemodynamic changes in various locations of a healthy male genital organ during visual sexual stimulation. The results also show the importance of sensor location on the genital organ for the resulting hemodynamic changes. We can foresee our results as a way for clinicians to obtain more accurate hemodynamic measurements from the penis, and also show the likelihood for NIRS enhanced diagnosis tool of male erectile dysfunction over the current standards.

## Background

Male erectile dysfunction (ED) is a disease that affects a large group of men and can have a severe negative effect on the subject’s life. ED has been found to affect 5-20% of the world’s male population [1]. ED is defined as the inability to achieve and maintain a penile erection sufficient enough to achieve satisfactory sexual performance and can have tremendous psychological health effects for the patient [2]. The inability to maintain a penile erection is biologically marked by the lack of blood flow to the penis. A normal penile erection is a result of many different dynamic, neural, and vascular interactions [3], therefore accurate measurements of penile hemodynamics is crucial in the diagnosis of erectile dysfunction.

There are many ways to assess the vascular functionality during penile erection including: selective pudendal angiography [4,5], duplex ultrasonography [6-9], and cavernosometry [9-11]. The issues with these assessments are the expense of the equipment, system complexity, and the fact that these tests require complete relaxation of the cavernous muscle. A common procedure to achieve muscle relaxation is the injection of an intracavernosal vasoactive agent [12,13]. This invasive procedure can be seen as a less than ideal approach of measuring ED due to the inconvenience for the subject. When conducting studies regarding erectile dysfunction and the genital organ in general, it is critical that the subject feels comfortable with the experiment’s paradigm. Any uncertainness about the paradigm may temporarily induce psychological erectile dysfunction, and may affect the final result [14].

Near Infrared Spectroscopy (NIRS) has shown to be a suitable sensor to detect the hemodynamic changes in the body under certain conditions. NIRS has been applied to detect hemodynamic changes in subjects with diseases ranging from epilepsy, Alzheimer’s, and including erectile dysfunction [15-19]. The compactness and convenience of NIRS systems make it an ideal sensor for observations of the genital organ hemodynamics during sexual stimulation. In the case of erectile dysfunction, the studies have largely been focused on the change in total blood volume and tissue oxygenation in the male genital organ [20,21]. However, to the best of our knowledge, there hasn’t been a thorough study showing the specific oxy- (HbO) and deoxy- (RHb) hemoglobin concentration changes and the effect of sensor location on the penile hemodynamics.

The aim of our study is to show that NIRS is a convenient, portable, and most importantly, a sensitive enough system to detect HbO and RHb changes during an erection of the genital organ. We also demonstrate that penile hemodynamics varies depending on the probe location on the genital organ. The dependency of penile hemodynamics according to NIRS probe location can be used to improve the diagnosis, treatment and overall understanding of male erectile dysfunction.

## Methods

### NIRS system

Our in house-built continuous wave NIRS probe consisted of one light emitting diodes (LED) (Epitex Inc. L735/850-40D32) and one monolithic photodiodes (Texas Instruments Inc. OPT101). The light emitting diode and photodiode was fixed onto the probe with a separation of 1 cm by means of a polydimethylsiloxane (PDMS) probe holder. The LED sequenced between the two wavelengths of 735 and 850 nm in order to obtain relative concentration changes of HbO and RHb. The sampling rate of the system was 2 Hz.

### Calculation of oxy-, deoxy-, and total hemoglobin concentration change

The detailed description of calculation of HbO, RHb, and HbT can be found in our previous report [22]. In the NIR range, we can assume that tissue background absorption is negligible and that HbO and RHb are the main chromophores in human tissue. Therefore, the change in optical density (ΔOD) at two wavelengths (735 and 850 nm) can be associated with the changes of HbO (ΔHbO) and RHb (ΔRHb) by

1 $$ \left[\begin{array}{c}\hfill \varDelta O{D}^{735}\hfill \\ {}\hfill \varDelta O{D}^{850}\hfill \end{array}\right]=\left[\begin{array}{cc}\hfill {\varepsilon}_{RHb}^{735}\hfill & \hfill {\varepsilon}_{HbO}^{735}\hfill \\ {}\hfill {\varepsilon}_{RHb}^{850}\hfill & \hfill {\varepsilon}_{HbO}^{850}\hfill \end{array}\right]\left[\begin{array}{c}\hfill \varDelta RHb\hfill \\ {}\hfill \varDelta HbO\hfill \end{array}\right]L $$

where $\varDelta OD = O{D}_{Transient} - O{D}_{Baseline},\ {\varepsilon}_{HbO}^{750},\ {\varepsilon}_{HbO}^{735},\ {\varepsilon}_{RHb}^{850},\ {\varepsilon}_{HbO}^{850}$ are extinction coefficients of RHb and HbO at wavelengths of 735 and 850 nm, *L* is an optical path length between the light source and detector.

In a scattering medium, *L* is not exactly equal to the source-detector separation, *d*, but rather approximated as *L* = *dDPF* (*DPF*: differential path length factor) [22]. If the scattering property of tissue is constant during the measurements, ΔHbO, ΔRHb, and ΔHbT can be obtained by

2 $$ \left[\begin{array}{c}\hfill \varDelta RHb\hfill \\ {}\hfill \varDelta HbO\hfill \end{array}\right]=\frac{1}{d\cdot DPF}{\left[\begin{array}{cc}\hfill {\varepsilon}_{RHb}^{735}\hfill & \hfill {\varepsilon}_{HbO}^{735}\hfill \\ {}\hfill {\varepsilon}_{RHb}^{850}\hfill & \hfill {\varepsilon}_{HbO}^{850}\hfill \end{array}\right]}^{-1}\left[\begin{array}{c}\hfill \varDelta O{D}^{735}\hfill \\ {}\hfill \varDelta O{D}^{850}\hfill \end{array}\right] $$

3 $$ \varDelta HbT=\varDelta RHb+\varDelta HbO $$

The raw data have been passed through Butterworth lowpass filter with cutoff frequency at 0.3 Hz, to eliminate physiological artifacts such as: heartbeat, respiration etc.

### Experimental setup

**Figure 1:**
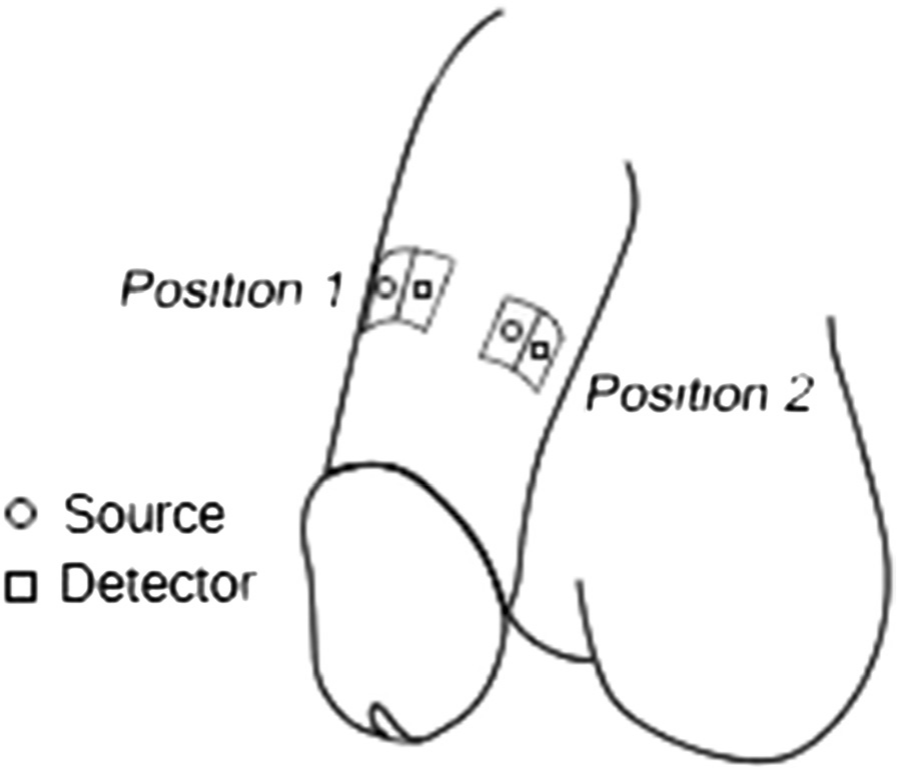
**NIRS Probe on genital organ.**

Our study consisted of three different placements of the probes, for three different experiments: a single probe placed on the side of the genital organ, a single probe placed on the top of the genital organ, and two identical probes placed on the top and side of the genital organ (Figure 1). The subject was the sole determinant of the exact location to place a probe on the genital organ, however received training beforehand of proper placement of the probe. The probe was affixed to the genital organ using commercial medical tape. A stable signal was confirmed by the subject and also by experiment administrator before the experiment began. The ability of the subject to attach the system himself demonstrated the overall convenience of the NIRS probe.

### Experiment paradigm

This study has been reviewed and approved by the Gwangju Institute of Science and Technology’s institutional review board (IRB 20140319-HR-10-02-02). The first two experiments were run for 6 healthy male subjects, age ranging from 28 ± 3 years old, with no previous history of ED. The informed written consent was obtained from each subject before the experiments. Each subject was placed in a dark experiment room with a chair and monitor. Before the experiment began, the administrator instructed the subject how to attach the probe. After that, the experiment was administered remotely by an experiment administrator in another room. A single probe was attached on the subject’s left-middle of the genital organ for the first experiment and a single probe was attached on the top and middle of the genital organ for the second experiment. (Figure 1) The first and second experiment involved the same subjects, but occurred on different days. As mentioned previously, the subject attached the probe and a stable signal was confirmed remotely by the administrator. The subject sat down and was shown two types of videos: a still image of a cross for one minute (baseline), and adult video (sexual stimulation) for two minutes, for a total of 4 minutes. (Table 1) The preference for adult video was obtained via questionnaire prior to the start of the experiment.

**Table 1:** **Sequence of videos**

***Type of video***	Baseline	Sexual video	Baseline
***Duration***	1 minute	2 minutes	1 minute

Because of the intriguing results that was collected from the first two experiments, a third experiment was design to simultaneously measure hemodynamic changes from the top and left middle of the genital organ three times, using just one subject, utilizing the same sequencing of videos. Two exact probes were designed with the same specifications, and affixed by the subject with commercial medical tape. This experiment was designed to eliminate the variability of individualistic factors such as interest in stimulation video and the individual condition of each subject.

## Results and discussion

The results section can be divided into two distinct sections. Our first set of results was acquired from six healthy male subjects with a probe placed on the left side (from the viewpoint of the subject) of the genital organ for one trial and a probe placed on top of the genital organ for another trial. The trials were independent of each other with appropriate rest in between and different visual sexual stimulations as to create the same sensation in the subject. The collection and post processing of the data occurred in entirely by using Matlab software.

**Figure 2:**
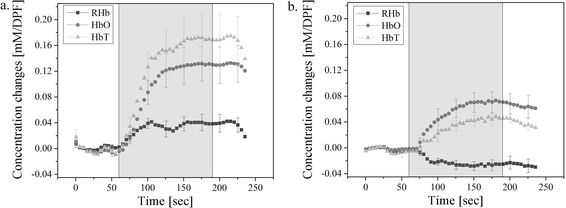
**The average hemodynamic changes from all subjects during. (a)** first experiment, a NIRS probe on the side; **(b)** second experiment, a NIRS probe on the top (error bars represents the standard errors from six subjects data).

Figure 2 shows the averaged HbO, RHb, and HbT concentration changes acquired from six subjects from the first two experiments. When the NIRS probe placed on the side of the genital organ, we can see from Figure 2a that HbO, RHb, and HbT concentration rises during the sexual stimulation period. Then, when the probe is placed on the top of the genital organ, Figure 2b indicates that the RHb decreases while HbO and HbT increases. Compared to Figure 2b, Figure 2a shows a much higher relative concentration changes for total hemoglobin.

**Figure 3:**
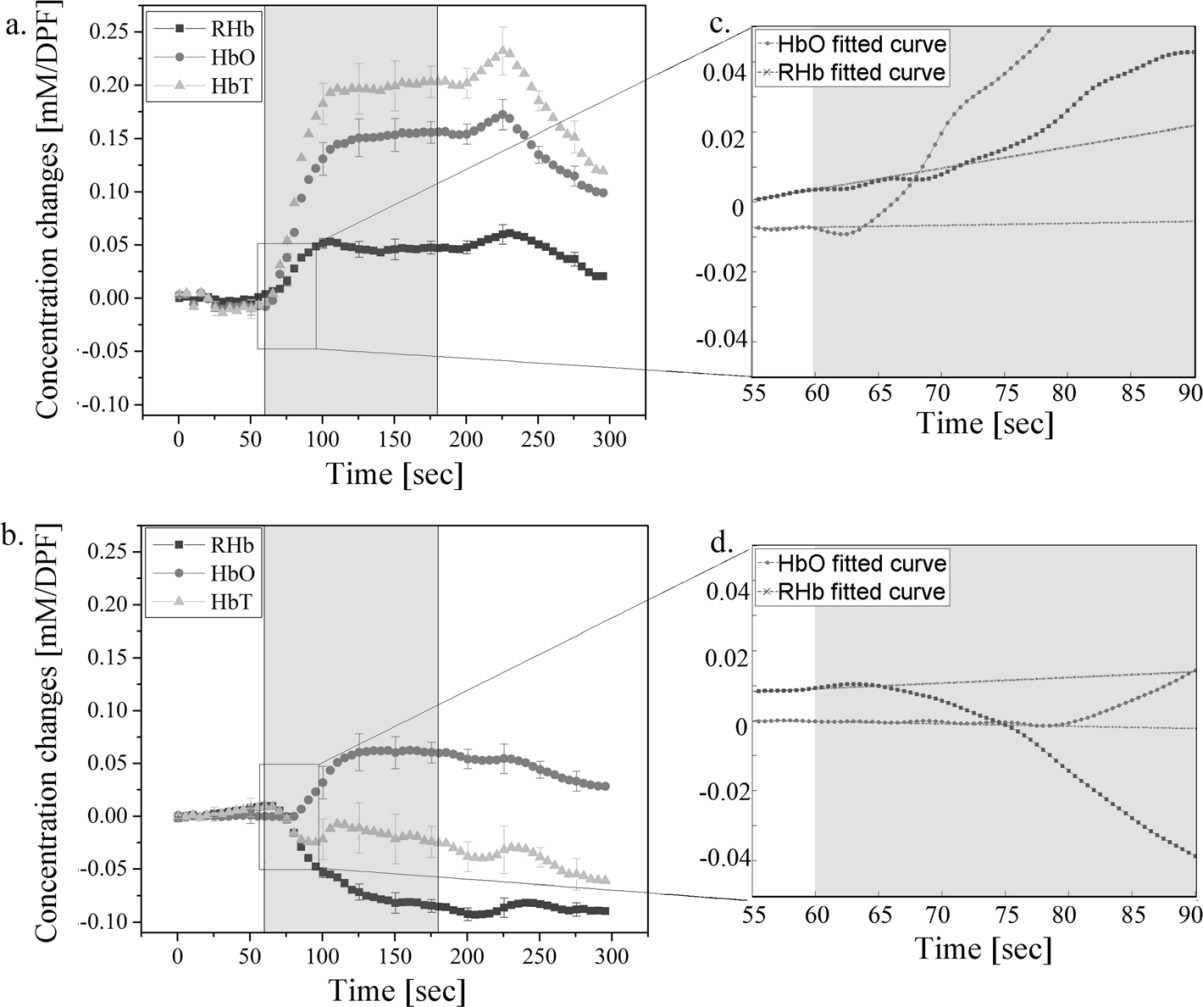
**The simultaneous measurement of hemoglobin concentration changes from one subject (averaged from three trials). (a)** a probe on the side; **(b)** a probe on the top; **(c)** and **(d)** are enlarged part of the signal obtained from side and top during the first 30 sec of stimulation, respectively (error bars represents the standard errors from three trials).

Figure 3 demonstrates the discrepancy of concentration changes of HbO, RHb, and HbT during penile erection between the top and the side of the genital organ simultaneously obtained from one subject. The resting stage after stimulation had been extended to one more additional minute due to subject's slow return back to a flaccid state. This proves the usefulness of NIRS as a real-time penile hemodynamic monitoring device. The figure shows the same pattern that has been observed from the two first experiments; an increase of HbO, RHb and HbT on the side of genital organ while the probe placed on the top showed not as substantial increase in HbO, a larger decrease in RHb concentration, and therefore a decrease in HbT.

In addition, the third experiment results show not only the different behavior of RHb but also the difference of response time between HbO and RHb occuring after the visual sexual stimulation. The probe placed on the side of genital organ showed that HbO change occurs before the change of RHb while the reversed response time between HbO and RHb was observed from the probe located on the top of the genital organ. (Figure 3c and d) This result implies the importance of multi-location measurement of penile hemodynamics in erectile dysfunction since it can provide distinct physiological process of penile hemodynamic from the genital organ during erection.

**Figure 4:**
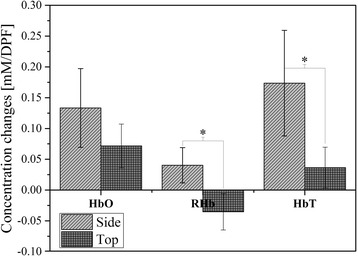
The level of concentration changes at the end of stimulus from the all experiments (error bars represents the standard errors from all three experiments) (* represents p < 0.05).

The changes of HbO, RHb and HbT concentration during the visual sexual stimulation are averaged from all three experiments and are compared between the top and the side of the genital organ. (Figure 4) HbO from the side probe showed greater increase than that from the top probe, and RHb from the side probe increased while it decreased from the top probe during the visual sexual stimulation. While the probe located on the side shows greater increase of HbT, the probe on the top showed much smaller increase or decrease of HbT depending on the amplitude between HbO and RHb. A Wilcoxon rank-sum test was performed between signals from the top and side, and it showed that RHb and HbT concentration changes during erection are significantly different between the side and the top of the genital organ (*p* < 0.05) while HbO concentration change did not show the statistical difference (*p* = 0.07).

**Figure 5:**
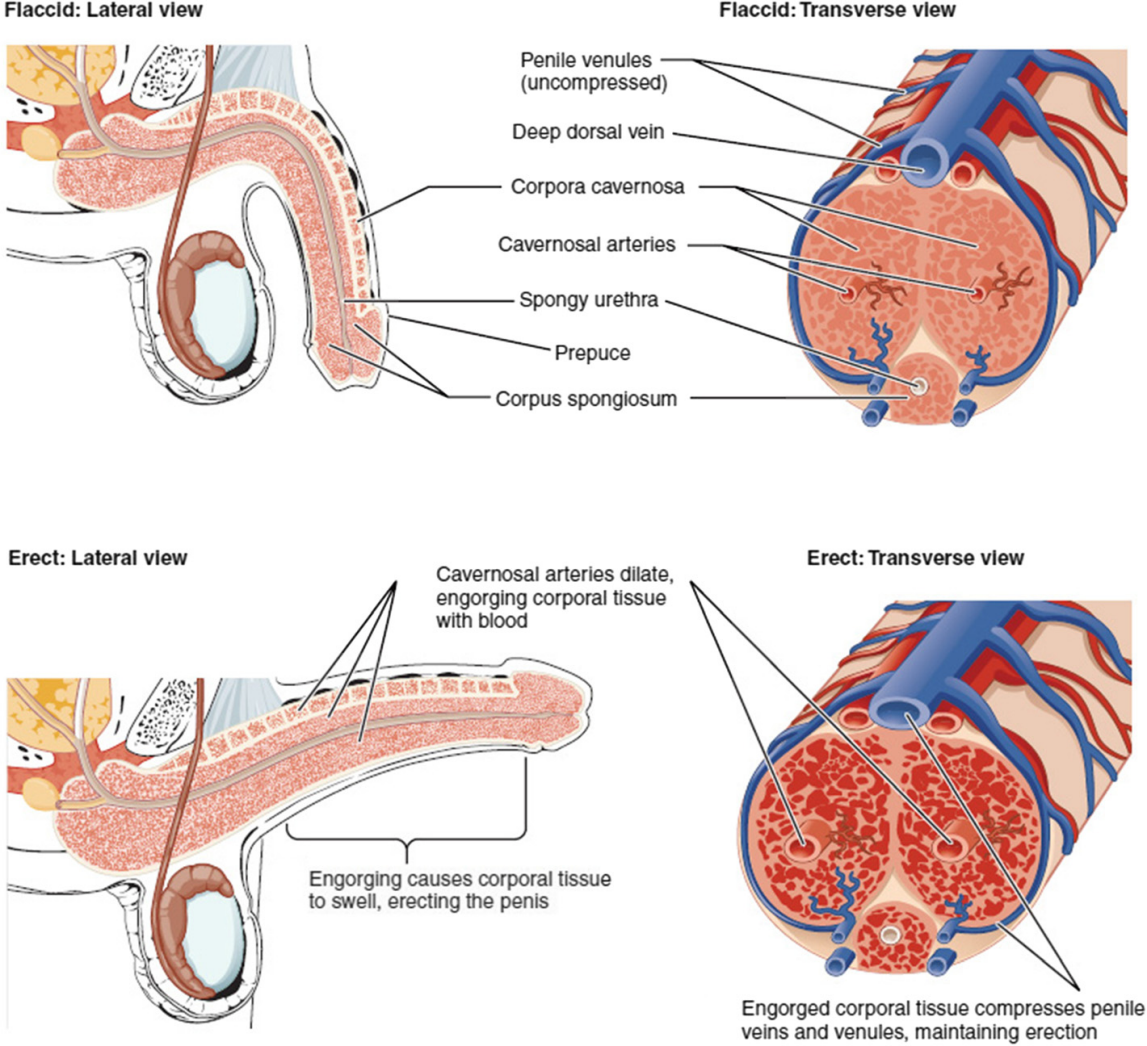
**The structural compartments of the penis during flaccid (top) and erected (bottom) state Image Source: Taken from Openstax College, 2014)** ***.***

Thereby, the results from the three experiments demonstrate that the distinction of physiological process occurs at different location during penile erection. The penile hemodynamics from the side of genital organ is similar to the hemodynamics from venous occlusion of the arm, which shows an increase of both HbO and RHb. This can be explained by understanding the process of erection as follows. Visual sexual stimulation causes a sexual arousal in brain which in turn releases the nitric oxide from nerve endings near blood vessels within the corpora cavernosa and spongiosum. This induces the dilation of penile arteries, and the resulting rapid increase of blood volume fills the corporal tissue. The increased blood volume in erectile chambers compresses the thin-walled penile venules which prevents the venous drainage of blood to maintain the erection. (Figure 5) Therefore, we see the increase of HbO due to the flow of arterial blood into corporal tissue while the compression to penile venules induces smaller increase of RHb.

Unlike the hemodynamics observed at the side of genital organ, the penile hemodynamics from the top of genital organ showed a decrease of RHb while HbO increased during visual sexual stimulation. The probe on the top of genital organ collects less signals from corporal cavernosa which is shown as a smaller increase of HbO than that from the side of genital organ. The decrease of RHb tells us that the expansion of corporal tissue caused less compression of dorsal vein due to its bigger diameter than the penile venules located on the side of genital organ so that there is less accumulation of venous blood but still with an increase of arterial blood on the top region of genital organ. All the results proved that NIRS is sensitive to monitor the penile hemodynamics during erection and multi-location measurements may tell us the details of erectile dysfunction and weaker erections.

## Conclusions

This preliminary study has shown strong indication that NIRS can be a very important device for the understanding of erectile dysfunction due to its ability to detect different hemodynamic changes in various locations of a healthy male genital organ during visual sexual stimulation. Our data set shows that different locations of the genital organ may show a substantial increase in HbO, RHb and HbT, or a rise of HbO and HbT, but a decrease in RHb during visual sexual stimulation. Especially in the case of the studies of the genital organ and erectile dysfunction, it is vastly important that the measurement system should be comfortable for the subject, while at the same time, provide sufficient and high quality data for researchers and clinicians alike. We hope that the unique insight gained by applying NIRS can be useful in the diagnosis of male erectile dysfunction and weak erection.

For our future studies, we hope to expand upon our findings in this paper to understand what other physiological changes the body undergoes during sexual stimulation. Since a healthy penile erection is a result of many complex psychological and physiological reactions, and the impairments in any of these reactions may cause ED, it is important to develop a system to observe these reactions. NIRS presents a realistic solution to conveniently observe the psychological and physiological changes that the body undergoes during sexual stimulation by using a single system. In turn, we would like to apply our system to the cases of erectile dysfunction patient to see if NIRS can observe different hemodynamic changes in ED cases than from the healthy subjects we have shown in this study. As it can be seen, NIRS gives a promising outlook for a biosensor that can greatly enhance the diagnosis and treatment of erectile dysfunction and weak erection.

## Abbreviations

ED: Erectile dysfunction
HbO: Oxy hemoglobin
HbT: Total hemoglobin
LED: Light emitting diodes
NIRS: Near infrared spectroscopy
PDMS: Polydimethylsiloxane
RHb: Deoxy hemoglobin
